# P20 Novel phenotypic, impedance-based fast antibiotic susceptibility test (AST) can distinguish between MRSA and MSSA in 2 h

**DOI:** 10.1093/jacamr/dlac133.024

**Published:** 2023-01-19

**Authors:** Bethany Martin

**Affiliations:** UK Health Security Agency, UK; University of Southampton, Southampton, UK

## Abstract

**Background:**

Antimicrobial resistance (AMR) is a global problem and is forecast that by 2050 it will cause 10 million deaths a year. Current antimicrobial susceptibility tests (ASTs) used clinically, can take 24–48 h or longer to report results, requiring that initial treatment uses broad spectrum antibiotics. The novel impedance-based fast AST (iFAST) method can report results within 2 h of exposure to an antibiotic. The impedance cytometer measures changes in the electrical charge flow between electrodes when bacterial cells flow individually through a microfluidic channel by an AC current of multiple frequencies. This is interpreted as a read-out of electrical radius (a surrogate measure of cell volume) and opacity (a measure of the resistance properties of the bacterial membranes). Exposure to antibiotics can change the electrical characteristics of the bacterial cell in size and opacity compared with the control sample. The number of exposed cells within a contour defined by a control sample, is used to assess how the cells following exposure.

**Objectives:**

To demonstrate concordance of this novel rapid method with the current gold standard AST, as performed in a diagnostic laboratory. To distinguish between MRSA and MSSA isolates. Cefoxitin was used as a surrogate for methicillin in laboratory testing as outlined in EUCAST guidelines. Sequentially collected bacterial isolates were used in the clinical microbiology laboratory of University Hospital Southampton from May–July 2022.

**Methods:**

60 blinded concurrent clinical isolates of *S. aureus* were taken from the clinical workflow and tested on the iFAST*. S. aureus* isolates were streaked onto blood plates and incubated at 37°C for 2 h. The bacteria were diluted into 3 mL of saline solution. The concentration of the bacteria was adjusted to 5 × 10^5^ cfu/mL, in line with EUCAST guidelines, exposed to the beta lactam antibiotic cefoxitin for 2 h at the EUCAST breakpoint concentration of 8 mg/L. Following exposure, the samples were diluted 1:20 in saline solution and measured on the iFAST. Cefoxitin-exposed cells were compared with a control sample, which has not been challenged with antibiotic. The iFAST results were compared with gold standard disk diffusion assay results, generated in the clinical lab as part of normal clinical service.

**Results:**

The iFAST results showed 100% concordance with disk diffusion susceptibility testing for cefoxitin carried out in parallel by the clinical laboratory. The data showed different electrical impedance changes for both resistant and susceptible isolates. Susceptible strains showed a decrease in electrical radius, suggesting that the cells are smaller and a significant reduction in overall cell count was observed. Resistant strains showed a distinctive increase in electrical radius, suggesting that the cells are larger in size when exposed to cefoxitin. However, this and other factors that may contribute to an increase in electrical radius are currently being investigated. An electrical MIC carried out for selected strains, was used to confirm that the transition from one response to the other corresponded to the MIC measured by microbroth dilution. An analysis of the data with hospital laboratory staff, suggested that the iFAST can provide an MRSA confirmation to clinicians 16 h before the current AST's, which could benefit patient management and treatment.

**Conclusions:**

The iFAST can rapidly differentiate between MRSA and MSSA isolates in concordance with current susceptibility testing in the clinical setting. The results help to show how the iFAST could reduce the time taken to provide critical and accurate antibiotic treatment to patients.

